# Therapeutic Use of Dendritic Cells to Promote the Extranodal Priming of Anti-Tumor Immunity

**DOI:** 10.3389/fimmu.2013.00388

**Published:** 2013-11-29

**Authors:** Lu Chen, Kellsye L. Fabian, Jennifer L. Taylor, Walter J. Storkus

**Affiliations:** ^1^Department of Immunology, University of Pittsburgh School of Medicine, Pittsburgh, PA, USA; ^2^Department of Dermatology, University of Pittsburgh School of Medicine, Pittsburgh, PA, USA; ^3^University of Pittsburgh Cancer Institute, Pittsburgh, PA, USA

**Keywords:** dendritic cells, extranodal, cross-priming, therapy, cancer

## Abstract

Ectopic lymphoid tissue, also known as tertiary lymphoid organs (TLO) develop adaptively within sites of chronic tissue inflammation, thereby allowing the host to efficiently crossprime specific immune effector cells within sites of disease. Recent evidence suggests that the presence of TLO in the tumor microenvironment (TME) predicts better overall survival. We will discuss the relevance of extranodal T cell priming within the TME as a means to effectively promote anti-tumor immunity and the strategic use of dendritic cell (DC)-based therapies to reinforce this clinically preferred process in the cancer-bearing host.

## Introduction

In the classical model of peripheral T cell activation, tissue-resident dendritic cells (DCs) capture antigens (such as foreign pathogens, tumor cell debris, etc.) in an inflammatory microenvironment, leading to the migration of antigen-laden CCR7^+^ DC to regional draining lymph nodes [LN; aka secondary lymphoid organs (SLO)], where activation of cognate T cells occurs (1–3). After appropriate proliferative expansion and maturation, T effector cells may then enter the blood circulation and be recruited into tissue sites where they are competent to recognize and react against relevant antigen-presenting cells, such as virally infected host cells or tumor cells (4). Recent evidence obtained in a range of translational and clinical models suggests, however, that this classical/conventional paradigm may be operationally overly simplistic, and that extranodal (cross)priming of antigen-specific T cells can occur in peripheral tissues, often times in conditionally established tertiary lymphoid organs (TLO) (5–9).

## SLO/TLO Development: Natural and Induced

The developmental formation of SLO is believed to require the interaction of so-called lymphoid tissue inducer cells (LTi) bearing a CD3^−^CD4^+^CD45^+^IL-7R^+^c-Kit^+^ phenotype that produce lymphotoxin α/β [LTα/β; Ref. (10, 11)] with LTβR^+^ stromal “organizer” cell populations that may derive from adipocyte precursors (12), leading to corollary stromal cell elaboration of the SLO homeostatic chemokines CCL19, CCL21, and CXCL13 (8, 9, 13–15). These chemokines sustain recruitment of LTi and other lymphocytes into SLO, resulting in the development of a mature lymphoid organ architecture [i.e., based on the formation of follicular structures containing B cells and surrounding “cortical” zones that are diffusely populated by CD4^+^ and CD8^+^ T cells, antigen-presenting cells (including CD11c^+^ DC), and PNAd^+^ high-endothelial venules (HEV; (8, 15–19))].

Naive (CD62L^+^CCR7^+^) T cells enter SLO via interaction with PNAd^+^ HEV which are “decorated” with the CCR7 ligand chemokines CCL19 and CCL21 on their luminal surface, thereby facilitating lymphocyte extravasation/directed motility from the blood into the lymph node (20). Of these two chemokines, CCL21 may play the more dominant role in recruiting naïve lymphocytes into SLO, while CCL19 may be differentially cytoprotective in sustaining nodal populations of lymphocytes (20–22). Prolonged CCR7-mediated signaling into recruited T cells, leads to intrinsic upregulation of the sphingosine-1 phosphate receptor 1, EDG1 (23), which is involved in the ultimate departure of primed T cell populations from SLO into the peripheral blood circulation (24, 25).

While classical SLO are encapsulated structures that develop in predictable locations as a consequence of normal immune system development, under pathologic conditions, ectopic lymphoid tissues (aka TLO) may develop in peripheral tissue sites of chronic inflammation (13, 26). TLO formation has been reported within inflamed organs of patients with rheumatoid arthritis (27–29), psoriatic arthritis (30), diabetes mellitus (31–33), autoimmune gastritis [AIG; Ref. (32)], juvenile dermatomyositis (34), and Sjögren’s syndrome (35), among others. TLO formation has also been identified in the lungs of influenza virus-infected mice (36), the livers of hepatitis C virus (HCV)-infected patients (37) and in the stomachs of patients infected with *Helicobacter pylori* (38). “Dysfunctional” human lung allografts exhibiting chronic inflammatory responses have also been found to commonly contain TLO (17).

Furthermore, a burgeoning literature supports tumor-associated TLO as important sites of extranodal T cell priming and epitope spreading in the responder T cell repertoire (13, 39). TLO have been identified in a subset of human melanoma lesions, in which mature DC were found to maintain intimate contact with recruited T cell populations, consistent with the notion of operational extranodal (cross)priming within the tumor microenvironment (TME) (40, 41). Similar results have been reported for murine melanoma models (7, 8). In line with this model, naïve lymphocytes have been identified in TLO within pulmonary lesions of patients with lung cancer, making it likely that these immune cells encounter their cognate antigen for the first time and develop into antigen-specific T effector cells within the TME *in vivo* (16, 42). TLO featuring DC/Type-1 T cell clusters proximal to B cell “nests” have also been identified in human non-small-cell lung cancer specimens (43). In such instances, the density of mature DC found in TLO appeared to be associated with improved long term survival (6, 43). In a subset of patients with breast cancer, HEV have been found in close proximity to LTβ^+^LAMP^+^ DC in association with profound B/T cell infiltrates in the TME and a more favorable clinical outcome (44). Furthermore, Mulé and colleagues have recently performed a metagene analysis on human (Stage IV, non-locoregional) melanoma metastases and identified a 12-chemokine gene signature (i.e., CCL2, CCL3, CCL4, CCL5, CCL8, CCL18, CCL19, CCL21, CXCL9, CXCL10, CXCL11, CXCL13) correlating with the presence of TLO (containing CD20^+^ B cell follicles with prominent areas of CD4^+^ and CD8^+^ T cells, but not FoxP3^+^ T_reg_ cells), with better overall survival noted in the TLO^+^ subset of patients (41). In a similar vein, Gu-Trantien et al. (45) have also recently observed that the presence of breast cancer infiltrating follicular CD4^+^ T helper cells (Tfh; expressing CD200, FBLN7, ICOS, SGPP2, SH2D1A, TIGIT, and PDCD1/PD-1, and producing the CXCL13 chemokine) may be directly correlated with; (i) the degree of tumor-infiltrating lymphocytes (TIL), (ii) the formation of TLO-like structures in cancer tissue, and (iii) improved patient clinical response to pre-operative chemotherapy and/or post-surgical disease-free survival.

The conditional formation of TLO in peripheral tissues appears to require the coordinate participation of a similar cast of cellular participants, soluble mediators, and signaling pathways associated with the orchestration of SLO development (14, 15). Ectopic delivery of LTα/β or LIGHT (aka TNFSF-14 or CD258) promotes PNAd^+^ HEV, CCL19/CCL21 production, massive naïve T cell infiltration, and (tumor-specific) cross-priming in the context of TLO structures (9, 18, 36, 46–49). For example, targeted therapeutic delivery of LTα into the TME via the administration of a fusion protein encompassing the LTα molecule linked to an antibody recognizing a tumor plasma membrane-associated disialoganglioside GD2 (i.e., ch14.18-LTα) resulted in slowed tumor progression and the establishment of mature TLO structures within 9 days of treatment initiation (8). The LTβR ligands LTα/β and LIGHT appear to act directly on endothelial cells and DC in activating NFκB and promoting the expression of adhesion molecules, such as PNAd, VCAM-1, E-selectin, and ICAM-1 by HEV and IL-12p70 production from DC (50–52). In particular, LIGHT is essential for DC-mediated cross-priming of antigen-specific Type-1 T cells (53). Indeed, ectopic expression of LIGHT in the TME elicits profound infiltration and cross-priming of naïve anti-tumor T cells in concert with upregulated stromal cell production of TLO-associated chemokines (CCL21, CXCL9, CXCL10, and CXCL13), increased expression of vascular adhesion molecules (MAdCAM-1, VCAM-1, PNAd), and the presence of mature DC within the TME (9). Interestingly, DC, natural killer (NK) cells, and even B cells can serve as LTα/β producers in pro-inflammatory environments, allowing for the establishment of an autocrine feed-forward loop promoting TLO development in peripheral tissues (36, 54–59). Consistent with these findings noted for pro-TLO immunobiology of LTβR ligands, blockade of the LTβR precludes formation of TLO *in vivo* (60).

In a similar manner, induced expression or ectopic delivery of LTβR downstream mediators, CCL19 or CCL21, in the TME results in inhibition of tumor growth or complete rejection of established tumors associated with increased infiltration by CD3^+^CCR7^+^ T cells and/or DCs in a range of cancer models (32, 61–70). Interestingly, these interventional maneuvers may also reduce frequencies of tumor-associated immunosuppressive T_reg_ cells and MDSC (61).

During the ontogeny of TLO in peripheral tissues, lymphatic vessels (i.e., PNAd^+^, MAdCAM-1^+^, LYVE-1^+^, and/or Prox-1^+^ HEV) and disorganized clusters of APC and infiltrating lymphocytes appear in advance of canonical mature lymphoid organ architecture characterized by B cell follicular regions (19, 71). Signals that instigate the diffuse-to-organized structural transition of TLO may be provided by cognate T cell recognition of relevant target cell populations within nascent TLO (15, 72). It is important to note, however, that immature TLO have been oft-associated with locoregional immune sequelae including manifestations of autoimmunity and anti-tumor efficacy (5, 32, 71). Hence, while mature TLO may ultimately develop in the chronic disease setting, clinical meaningful immunobiology occurs in advance of such structural developments.

## Therapeutic Promotion of TLO

If the formation of TLO allows for extranodal (cross)priming of antigen-specific T cells that are linked to disease pathogenesis (i.e., autoimmunity) or resolution (i.e., infectious disease, cancer), then means by which to prevent or enhance TLO development, respectively, in affected tissues would be anticipated to impact clinical outcome. Perhaps the most strategically simple means by which to apply this paradigm in the cancer setting reflects the implantation of SLO/TLO directly into the TME. Recently, scaffold-based lymphoid tissue engineering has been developed as a means to transplant “intact” TLO directly into tumors in order to affect clinical benefit (73). A previously mentioned alternative to this strategy is clearly the delivery of the LTβR ligands LTα, LTβ, or LIGHT, agonist anti-LTβR antibodies or downstream TLO-associated chemokines (CCL19, CCL21, CXCL13) via protein-based or genetic therapy in order to instigate the locoregional development of TLO in the TME leading to inhibition of tumor growth *in vivo* and extended overall survival (8, 9, 48, 74, 75).

## Use of DC-Based Therapy to Promote Extranodal Priming of Anti-Tumor T Cells

It also appears that the administration of appropriately activated/engineered DC is sufficient to nucleate and/or maintain the development of TLO *in vivo* (36, 72). For instance, mice vaccinated sub-cutaneously with syngenic DC loaded with apoptotic/necrotic B16 melanoma cell debris develop operational TLO [pseudocapsule; PNAd^+^ vascular endothelial cells (VEC), T cell/DC infiltrates] at sites of injection, leading to the activation of protective anti-tumor immunity (72). DC genetically engineered to secrete high-levels of CCL21 (DC.CCL21) and injected directly into B16 murine melanomas promote strong extranodal T cell cross-priming/recruitment into the TME, even in LTα −/− mice that lack SLO (8, 68). The superiority of DC.CCL21 in enhancing the cross-priming of protective Type-1 anti-tumor T cell responses has also been confirmed in alternate murine models (76, 77).

**Figure 1:**
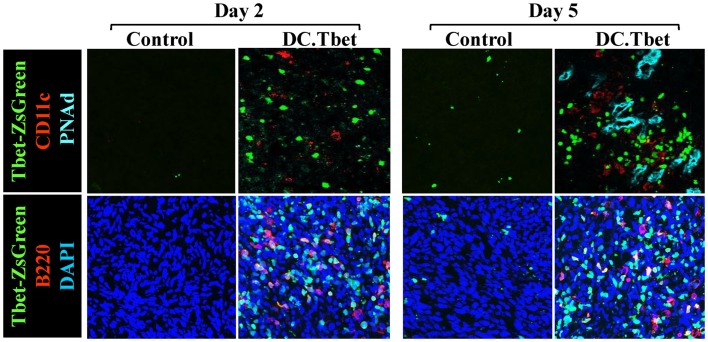
**Intratumoral administration of dendritic cells engineered to express T-bet/TBX21 (DC.Tbet) promote the rapid infiltration of Type-1-polarized lymphocytes and dendritic cells and the development of PNAd^+^ endothelial cells (i.e., HEV)**. Tbet-ZsGreen Tg mice were injected sub-cutaneously with syngenic MCA205 sarcoma cells and tumors allowed to progressively grow for 7 days, at which time control DC (Control) or DC engineered with recombinant adenovirus to express murine T-bet cDNA were inoculated directly into tumors, as previously described (5). Two or 5 days after DC injection, the mice were euthanized and tumor sections evaluated by fluorescence microscopy for expression of Tbet-ZsGreen protein, CD11c (a marker of DC), B220 (a B cell marker), and PNAd (i.e., Peripheral lymph Node Addressin; a high endothelial venule (HEV) cell marker). PNAd^+^ HEV were not observed by 2 days post-treatment, but became prevalent by 5 days post-injection of DC.Tbet cells. B, T, and NK cell infiltrates into DC.Tbet [Figure 1 and (5)] exhibited a diffuse distribution pattern in day 2 and day 5 DC.Tbet-treated tumors. Type-1 polarity in infiltrating cells is denoted by nuclear expression of Tbet-ZsGreen. Data are representative of images obtained in three independent experiments performed.

**Figure 2:**
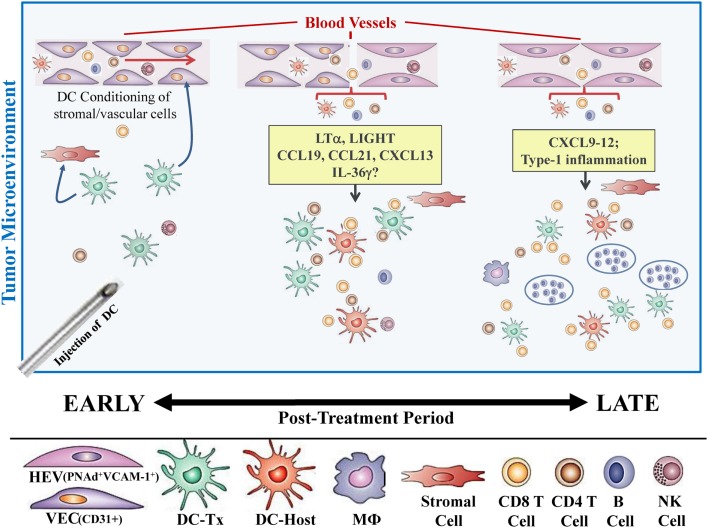
**Hypothetical paradigm for extranodal priming of T cells after intratumoral administration of DC.Tbet cells**. Injection of DC.Tbet (but not control DC) into the TME leads to the conditioning of tumor-associated stromal cells and vascular endothelial cells (VEC), resulting in stromal cell production of chemokines recruiting naïve leukocytes (B, T, NK cells) and VEC expression of adhesion molecules, such as VCAM-1, as early as day 2 post-treatment [Figure 1 and (5)]. Recruited lymphocytes are assembled in diffuse patterns around CD11c^+^ (both injected and host) DC and have already acquired Type-1 functional polarization, based on expression of the Tbet reporter protein (Tbet-ZsGreen) *in vivo*. PNAd^+^ HEV are not formally required for early recruitment of naïve T cells into the TME since these structures do not become discernible until later time points [i.e., day 5; Figure 1 and (5)]. B220^+^ B cells recruited into the TME as a consequence of treatment with DC.Tbet cells are not organized into follicle-like structures during the day 2–5 time period, but may become organized in this manner at even later time points (i.e., ≥day 9 post-therapy), based on previous reports employing alternate immunotherapeutic interventions, such as ch14.18-LTα (8). While therapeutic benefits in our model were largely T cell-dependent and detectable prior to the establishment of formal TLO structures (based on the development of B cell follicles), the presence of “mature” TLO in human tumors has been associated with better clinical prognosis.

In our recent paper (5), we noted that DC engineered to express the Type-1 transactivator protein T-bet (DC.Tbet) and injected directly into sarcomas growing progressively in C57BL/6 mice, led to the cross-priming of protective immunity that was independent of host CD11c^+^ or BATF3^+^ DC or the ability of the injected DC.Tbet to migrate to SLO. Instead, we detected the rapid recruitment of NK cells and naïve T cells into the TME within 48 h of DC.Tbet administration, and the differentiation of these TIL into Type-1 effector cells *in situ* within the TME. As shown in Figure 1, we observed a diffuse but interactive collection of CD11c^+^ DC and Tbet^+^ cells [including both T cells (5) and B220^+^ B cells] within the TME of MCA205 sarcomas by 48 h post-treatment with DC.Tbet, but not control DC. PNAd^+^ HEV were not evident at this early time point, but were readily imaged in proximity to large DC-Tbet^+^ lymphocyte clusters by 5 days post-treatment with DC.Tbet (but not control DC). These data suggest that extranodal priming of protective immunity using therapeutic DC delivery occurs in advance of the formal adoption of classical TLO anatomic structures within the TME (Figure 2), and that indeed, the development of such Type-1 cognate immunity (and its pro-inflammatory signals) in the TME may be required for subsequent evolution of mature TLO formatting, as described by Schrama et al. (8). Interestingly, a gene array analysis of DC.Tbet versus control DC did not reveal any striking differences in expression of LTA, LTB, LIGHT, CCL19, CCL21, or CXCL13 mRNA transcripts, suggesting a potentially novel mechanism associated with early TLO development triggered by this DC-based therapy [(5) and Chen, unpublished data]. In this regard, we noted a striking enhancement in DC.Tbet production of IL-36γ/IL-1F9 (>34-fold; Chen, unpublished data). IL-36γ is a novel IL-1 family member cytokine that has been previously reported to be a potent recruiter and activator of naïve T cells that develop strong Type-1 functional polarity (78, 79). As in the case of LTβR ligands, IL-36 also triggers NFκB activation in IL-36R^+^ DC (79–82), which may prove pivotal for autocrine potentiation of Type-1 DC function and a pro-TLO TME. Whether tumor-associated VEC express IL-36R and activated NFκb in response to IL-36 remains an unanswered question. We are currently evaluating the impact of IL-36γ knock-down in DC.Tbet in order to determine the direct relevance of IL-36γ in the development of TLO and protective immunity in the TME of mice treated with intratumoral administration of DC.Tbet.

## Summary and Future Perspectives

In the cancer setting, the ability of the host to develop ectopic lymphoid organs (TLO) within or proximal to sites of active disease appears to represent a positive prognostic factor for overall patient survival. TLO represent a regional “factory” in which naïve T cells (and B cells) may be cross-primed by tumor-resident antigen-presenting cells, such as DC, leading to poly-specific adaptive immunity that may limit disease progression and conceivably metastatic spread. By limiting the need for antigen-loaded DC to migrate to tumor-draining SLO, and the corollary requirement of SLO-primed T cells to be recruited back into tumor sites, TLO may operationally increase the efficiency of anti-tumor T cell cross-priming and the therapeutic action of such T effector cells in the TME. Translational studies clearly suggest that TLO formation in the TME may be therapeutically fostered by the directed delivery of LTβR ligands in both protein- and gene-based formats. At present, LTβR agonist-based therapies are in their infancy with only rhLTα thus far evaluated in phase I clinical trials, where minimal anti-tumor efficacy was observed in patients with melanoma or renal cell carcinoma (83). The inability to focus this potent TLO induction agent in appropriate sites of disease in order to most effectively recruit and activate protective immunity in treated patients must be considered a possible limitation to the current treatment strategy. The ligation of rhLTα to a cancer-specific antibody or the intratumoral administration of this agent could improve anti-tumor efficacy and coordinately reduce current off-target toxicities [i.e., grade III chill, grade III fever, and grade III dyspnea; Ref. (83)].

Improved targeted delivery of LTβR ligand or downstream chemokine gene therapies is conceptually attractive given pre-clinical results in murine tumor models. To date, however, only a recombinant adenovirus encoding hCCL21 has been developed for phase I clinical application, with the intent to deliver rAd.CCL21-infected patient DC directly into tumors in patients with late stage human lung cancer (84) or in vaccine formulations applied to patients with melanoma (85). Although this approach requires further optimization of the clinical vector based on low levels of CCL21 produced by engineered DC, melanoma patients treated at the lowest dose tier of DC.CCL21 did develop lymph node-like structures based on immunohistochemical analysis of vaccination site biopsies (James Mulé, personal communication). Our own pre-clinical data would support the clinical application of DC.Tbet directly into accessible tumor lesions as a means to drive TLO development and protective immunity in the TME. Motivation for the development of prospective phase I trials using DC.Tbet cells will be intensified when the underlying mechanism of action for this treatment modality has been defined.

Finally, in a related note, antagonists of LTβR ligands (such as BTLA and CD160) have been shown to be immunosuppressive molecules in inhibiting DC homeostasis as well as the protective effector functions mediated by T cells and NK cells (74, 86–90). It is therefore conceivable that endogenous levels of TLO development and corollary anti-tumor immunity may be bolstered therapeutically as a consequence of administering agents (i.e., antagonist antibodies or DC genetically engineered to produce specific inhibitors locoregionally in the TME) that are capable of blocking the action of BTLA or CD160 *in vivo*.

## Conflict of Interest Statement

The authors, editor and chief editor declare that while the author Walter Storkus and the editor Lisa Butterfield are currently employed by the same institution, University of Pittsburgh, USA, there has been no conflict of interest during the review and handling of this manuscript, and the manuscript reviewers involved were from other institutions.
